# The DNA secondary structures at telomeres and genome instability

**DOI:** 10.1186/s13578-020-00409-z

**Published:** 2020-03-26

**Authors:** Jun Tan, Li Lan

**Affiliations:** 1 Harvard Medical School, Massachusetts General Hospital Cancer Center, Charlestown, MA 02129 USA; 2 Department of Radiation Oncology, Harvard Medical School, Massachusetts General Hospital, Boston, MA 02115 USA

**Keywords:** R-loop, G-quadruplexes, Transcription, Telomere, Therapy, Secondary structures

## Abstract

Telomeric DNA are TTAGGG tandem repeats, which are susceptible for oxidative DNA damage and hotspot regions for formation of DNA secondary structures such as t-loop, D-loop, G-quadruplexes (G4), and R-loop. In the past two decades, unique DNA or RNA secondary structures at telomeres or some specific regions of genome have become promising therapeutic targets. G-quadruplex and R-loops at telomeres or transcribed regions of genome have been considered as the potential targets for cancer therapy. Here we discuss the potentials to target the secondary structures (G4s and R-loops) in genome as therapy approaches.

## Background

**Fig. 1:**
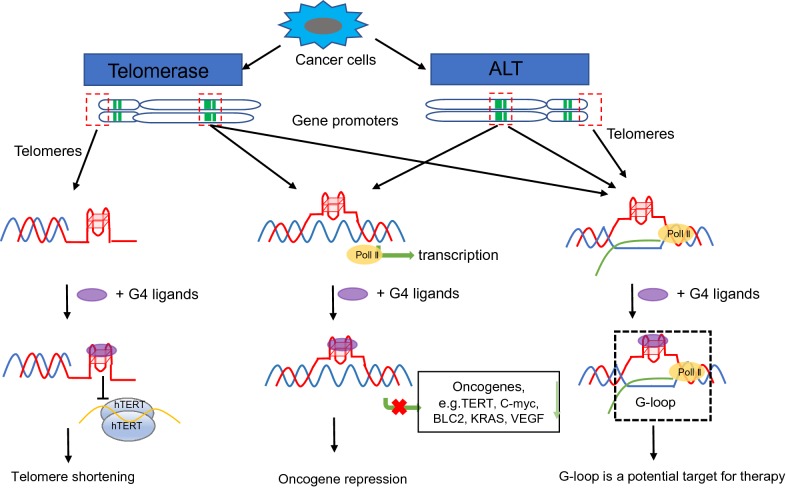
Targeting G-quadruplex structures in telomerase positive or ALT cancer cells. Left: unresolved G4s block the function of telomerase for telomere elongation in telomerase positive cells; Middle: stabilized G4s at some oncogene promoters lead to the repression of those oncogenes; Right: Targeting G-loop with G4s ligands or R-loop inhibitors may be a potential therapeutics for ALT cancers

Telomeres are nucleoprotein structures at the end of each chromosome, which protects the end of the chromosome from deterioration or from fusion with chromosomes, and the hotspot region for formation of many secondary nucleotide structures [1, 2]. Cancer cells get the ability to conquer the replication problem via either telomerase or Alternative Lengthening of Telomeres (ALT) pathway, which involves recombinational mechanisms to overcome incomplete replication of telomeres [3–5] (Fig. 1). Telomeric DNA are TTAGGG tandem repeats, which are susceptible for oxidative DNA damage and hotspot regions for formation of DNA secondary structures such as t-loop, D-loop, G-quadruplex (G4), and R-loop [6–8]. Single strand G-rich overhang folds back and invades into the double-stranded telomere tract to form a T-loop and D-loop structure, protecting the end of chromosome from being recognize as double strands breaks (DSBs) [9–11]. When single-stranded guanine-rich DNA sequences fold into stable intramolecular and intermolecular four-stranded non-B DNA structures, such a structure is so called G-quadruplexes, which may play important roles in the regulation of gene expression, DNA repair, epigenetic regulation and telomere biology [12–14]. R-loops are the three-stranded nucleic acid structure that contains a DNA: RNA hybrid and displaced DNA strand. We recently discovered that ROS-induced DNA damage at telomeres triggers R-loop accumulation in a TERRA and TRF2-dependent manner [8].

Since Last century cancer chemotherapy gained the success relying on highly cytotoxic drugs, which directly or indirectly disrupt the transcription and/or replication of cellular DNA in both normal cells and tumor cells [15]. To avoid the side effects of these toxic agents, investigators have paid their attentions to design and select the more selective drugs through many strategies. In the past two decades, unique DNA or RNA structures at telomeres or some specific regions of genome have become promising therapeutic targets. The G4 and R-loop structures at telomeres or transcribed regions of genome have been considered as the potential targets for cancer therapy. To date, several strategies that target these specific structures or proteins involving maintaining these structures have been developed by a number of laboratories. Here, we summarize some recent studies that aimed at targeting the secondary structures (e.g. G4 and R-loop) as therapeutic approaches for killing cancer cells.

### The distribution of G-quadruplex structures in genome

In 1962, Gellert.et al. first reported the G-tetrad structure in guanylic acid with an X-ray diffraction study [16]. G4 antibodies were made to detect those structures within genomic DNA. Schaffitzel et al. firstly used a high-affinity single-chain antibody to detect quadruplex structure at the telomere of ciliate Stylonychia lemonade [17]. Since then several G4 antibodies have been generated by a few groups to visualize those structures in cells, including Sty49 [18], BG4 [19] and 1H6 [20]. With the combination of next-generation sequencing and genomic mapping, several groups have revealed that the distribution of unique sequences that possess the potential to form stable G4 structure. The human genome contains 376,000 potential G4 structures [21, 22]. The potential G4 structures are highly occurrent in telomeres and promoter regions of oncogenes [23, 24]. Besides telomeres and promoter regions, ribosomal DNA [25], 5′untranslated region of mRNA [26], or TERRA [27] are also potent to form the G4 structures.

### *Targeting* G-quadruplex structures of hTERT

From the genome-wide sequence analyses, potential G4 structures are enriched in promoter regions, which span 1 kb upstream of the transcription start site in humans and other vertebrates genes [24, 28]. Previous studies have paid great attentions to cancer-related genes, e.g. hTERT, c-MYC [29], BLC2 [30], KRAS [31, 32], and VEGF [33], which contain enriched G4 motifs in their promoter regions. A number of G4-targeting ligands have been developed to target the promoters of these genes as potential biomedical targets for antitumor therapy. One major obstacle impeding the clinical application of G4 ligands is the lack of selectivity. Recently, people have entered a new phase of the development of next-generation ligands that interact with G4. The goal is to improve the ligand selectivity to a particular G4 to be targeted, potentially leading to the development of molecules with high antitumor activity and bioactivity with minimal antitumor therapy side effects.

Elevated hTERT expression is observed in ~ 90% of human cancer cells, whereas it is normally silenced in most normal cells. Therefore, hTERT has been considered as the most attractive biomedical target for cancer treatment. Investigators have used two approaches to suppress the function of hTERT: downregulate the expression of hTERT or inhibit the activity of hTERT. Some unselective G4 ligands, such as telomestatin [34, 35] and substituted acridines [36, 37], may bind to a large scale of G4 structures, including hTERT, c-kit, KRAS or c-MYC promoters (Fig. 1 middle). Those unselective ligands may cause bunch of side effects along with their cancer therapeutic effects, which limited their clinic applications. Recently, Hurley and colleagues used a unique approach to address the issue of hTERT downregulation on the basis of the mutations in a G4-mediated manner. They have developed a small molecule (GTC365) that acts at an early step in the G4 folding pathway to redirect mutant promoter G-quadruplex misfolding and reduce hTERT activity through transcriptional repression. They also demonstrate the selectively therapeutic potential of this strategy in melanoma cells that overexpress hTERT [38]. In addition to hTERT, some G4 ligands that more selectivity target to particular G4s at the promoter of specific cancer-related genes (e.g. C-MYC, BLC2, KRAS, and VEGF) have been reported. Tan and colleagues report a new four-leaf-clover-like molecule, IZCZ-3, that have about eightfold preference for the c-MYC over the G4s in the promoters for other genes. More importantly, this ligand showed cytotoxicity against cancer cell lines overexpressing c-MYC but not against normal cells, suggesting reduced side effects based on G4 selectivity on c-MYC [39]. Other ligands, like Furo[2,3-d]pyridazin-4(5H)-one 9 (BLC2) [40], Indolo[3,2-c]quinolines (IQc) (KRAS) [41], and SYUIQ-FM05 (VEGF) [42], has also been reported. Those findings shed a light on the developing of the next-generation G4s ligands, which have high antitumor activity and bioactivity with minimal side effects.

### Targeting G-quadruplex structures at telomeres

The regions of eukaryotic genomes with the highest concentration of potential G4 structures are telomeres [21, 23]. Telomeric G4 structures have been considered as attractive anticancer targets for many years. The investigators have successfully developed a large number of compounds that targeting telomere–G-quadruplexes [43] after the first G4s ligands (2,6-diamidoanthraquinone) [44] being reported. In telomerase positive cells, the G-overhang is extended by telomerase, a reverse transcriptase enzyme carrying its own RNA template (Fig. 1 left). G4s ligands bind to G4s tightly and block the telomerase activity through disrupting the base-pairing between G-overhang and telomerase RNA [12, 45, 46].

A lot of efforts were devoted to design more selectable G4s ligands that adopted at telomere 3′ overhang region these years. Some enantiomers, such as Ni–P, exhibit an ability to convert a monomeric antiparallel form to a monomeric hybrid form, and inhibit the cell growth via disputing the localization of TRF2 and POT1 at telomeres [47, 48]. Then, several studies found that a large variety of alternative higher-order structures derived from the canonical telomere G4 might be adopted at the 3′- overhang region. Thus, the unique structure and motif of these ligands are amenable to the gain of specificity for telomere G4s [49–52].

### Telomeric R-loop formation and its relevance with G4 structure

The overabundance of R-loop as shown in a number of neurological syndromes and cancer [53, 54]. The out balance of R-loop leads to genome instability and replication stress, which is a molecular symptom of tumor cells [55]. Therefore, targeting R-loop has been considered as a potential approach to sensitize certain tumors to chemotherapeutic treatment. In 1997, Weaver et al. reported that F8-actinomycin D exhibits a unique selectivity against leukemia cells [56]. However, DNA:RNA hybrids are often formed at transcribed genome, the application of those molecules that directly bind to DNA:RNA hybrids was limited. Recently, researchers turned to pay more attentions on targeting R-loop binding proteins. Andrés Aguilera and colleagues reported that trabectedin and lurbinectedin induced DNA-RNA hybrids-dependent DNA damage in HeLa cells, impairs DNA replication and causes genome instability. The high level of R-loops increases cell sensitivity to those antitumor drugs [57]. Especially, some homologous recombination deficiency cancer cells, which showed the elevated level of R-loop formation, were hypersensitive to genotoxic drugs such as etoposide, camptothecin, trabectedin and PARP inhibitors [58–60]. Furthermore, several other compounds have also been reported to increase R-loops, including topoisomerase1 inhibitors [61], spliceosome inhibitors [62, 63], and RNase H2 inhibitors [64, 65].

G4 structures form in a similar genomic context as R-loops. Recent studies indicate the presence of co-existence of R-loop and G4, known as “G-loop”, which is a unique structure where G4 is formed at the displaced single-stranded of an R-loop, both in vivo and in vitro [66]. In 2004, Nancy Maizels and Colleague first described the formation of G-loop in vitro and in Escherichia coli using electron microscopy. Recently, several studies revealed that G4 ligands (PDS, CX-5461) induce R-loop-mediated DNA damage and cell death in cancer cells [67, 68]. Giovanni Capranico et al. used different G4 ligands, including pyridostatin, Braco-19, and FG, to stabilize G4 structures and simultaneously increase R-loop levels within minutes in human cancer cells. The increased level of R-loop leads to the accumulation of γH2AX foci and of G2/M cells, which are both hallmarks of genomic DSBs and DNA damage response. Importantly, overexpression of an exogenous human RNaseH1 rescued DNA damage induced by G4 ligands in BRCA2-depleted cancer cells, which indicated the G4 ligands can induce DNA damage by an R loop-dependent mechanism [67].

### Is the telomeric R-loop structure a potential target for cancer therapy?

R-loops play an import role in telomere maintenance in telomerase negative cancer cells. In ALT cancer cells, R-loop facilitated telomere elongation through a recombination mechanism. Arora et al. first showed that the RNA endonuclease RNaseH1 regulates the levels of RNA–DNA hybrids between telomeric DNA and the long noncoding RNA TERRA, and is a key mediator of telomere maintenance in ALT cells. Then, several studies also confirm the function of R-loop in telomere maintenance [69, 70]. Our group recently reported that the R-loop-CSB-RAD52-POLD3 axis contributes to the repair of Reactive oxygen species (ROS) induced telomeric damage in ALT cancer [8]. Target R-loop interacting proteins at genome are also possible to enhance the cell killing effects. Therefore, targeting telomeric R-loop could be the potential treatments for ALT tumors, with more studies revealed the molecular details of ALT and the mechanisms involved in its engagement (Fig. 1 right).

There are a lot of reasons that the application of telomerase inhibitors in cancer therapy has not got too much progress. Although the non-selectivity of those inhibitors may the major limitation for their application, other reasons cannot be excluded. Two of those reasons are that the probability co-existence of telomerase and ALT pathway in some specific tumor [71–74], and ALT pathway may be activated after suppressing telomerase by inhibitors [75]. The information about the activation of ALT pathway in telomerase-positive cancer cells that treated with telomerase inhibitors is limited. Combined strategies that target both telomerase and ALT could be proved to be a powerful approach for the treatment of such tumors.

## Conclusions

Both G4 and R-loops structures are potential targets for cancer therapy, however, questions including how to improve the selectivity of drugs, how to reduce the resistance and side effects, are the major obstacles for their future application. Actually, several clinical trials of G4 ligands were withdrawn due to these reasons. More ligands that have higher affinities to spatial conformations of G4 structures within unique regions of genomes are expected in the future.

## Abbreviations

G4: G-quadruplexes
ALT: Alternative Lengthening of Telomeres
DSBs: Double Strands Breaks
ROS: Reactive oxygen species
